# Long-term effect of mobile phone use on sleep quality: Results from the cohort study of mobile phone use and health (COSMOS)

**DOI:** 10.1016/j.envint.2020.105687

**Published:** 2020-07

**Authors:** Giorgio Tettamanti, Anssi Auvinen, Torbjörn Åkerstedt, Katja Kojo, Anders Ahlbom, Sirpa Heinävaara, Paul Elliott, Joachim Schüz, Isabelle Deltour, Hans Kromhout, Mireille B. Toledano, Aslak Harbo Poulsen, Christoffer Johansen, Roel Vermeulen, Maria Feychting, Lena Hillert, the COSMOS Study Group

**Affiliations:** a Unit of Epidemiology, Institute of Environmental Medicine, Karolinska Institutet, Stockholm, Sweden; b STUK – Radiation and Nuclear Safety Authority, Environmental Radiation Surveillance and Emergency Preparedness, Helsinki, Finland; c Tampere University, Faculty of Social Sciences/Health Sciences, Tampere, Finland; d Department of Clinical Neuroscience, Karolinska Institutet, Stockholm, Sweden; e Department of Stress Research, Stockholm University, Stockholm, Sweden; f Finnish Cancer Registry, Helsinki, Finland; g Department of Epidemiology and Biostatistics, School of Public Health, Imperial College London, London, UK; h Medical Research Council (MRC) Centre for Environment and Health, School of Public Health, Imperial College London, London, UK; i National Institute for Health Research (NIHR) Health Protection Research Unit on Health Impact of Environmental Hazards, Imperial College London, London, UK; j NIHR Imperial Biomedical Research Centre, Imperial College London, London, UK; k International Agency for Research on Cancer, Environment and Radiation Section, Lyon, France; l University of Utrecht, Institute for Risk Assessment Sciences, Utrecht, the Netherlands; m Danish Cancer Society Research Center, Copenhagen, Denmark; n CASTLE Cancer Late Effect Research Oncology Clinic, Center for Surgery and Cancer, Rigshospitalet, Copenhagen, Denmark; o Centre for Occupational and Environmental Medicine, Stockholm County Council, Stockholm, Sweden; p Unit of Occupational Medicine, Institute of Environmental Medicine, Karolinska Institutet, Stockholm, Sweden

**Keywords:** Cohort study, Cell phone, Sleep disturbance, Insomnia, Electromagnetic fields, CI, confidence interval, COSMOS, Cohort Study of Mobile Phone Use and Health, GSM, 2G, Global System for Mobile Communications, MOS, Medical Outcome Study, OR, Odds ratio, REM sleep, Rapid eye movement sleep, RF-EMF, Radiofrequency electromagnetic field, SF-12, 12-Item Short Form Health Survey, UMTS, 3G, Universal Mobile Telecommunications Service

## Abstract

**Background:**

Effects of radiofrequency electromagnetic field exposure (RF-EMF) from mobile phone use on sleep quality has mainly been investigated in cross-sectional studies. The few previous prospective cohort studies found no or inconsistent associations, but had limited statistical power and short follow-up. In this large prospective cohort study, our aim was to estimate the effect of RF-EMF from mobile phone use on different sleep outcomes.

**Materials and methods:**

The study included Swedish (n = 21,049) and Finnish (n = 3120) participants enrolled in the Cohort Study of Mobile Phone Use and Health (COSMOS) with information about operator-recorded mobile phone use at baseline and sleep outcomes both at baseline and at the 4-year follow-up. Sleep disturbance, sleep adequacy, daytime somnolence, sleep latency, and insomnia were assessed using the Medical Outcome Study (MOS) sleep questionnaire.

**Results:**

Operator-recorded mobile phone use at baseline was not associated with most of the sleep outcomes. For insomnia, an odds ratio (OR) of 1.24, 95% CI 1.03–1.51 was observed in the highest decile of mobile phone call-time (>258 min/week). With weights assigned to call-time to account for the lower RF-EMF exposure from Universal Mobile Telecommunications Service (UMTS, 3G) than from Global System for Mobile Communications (GSM, 2G) the OR was 1.09 (95% CI 0.89–1.33) in the highest call-time decile.

**Conclusion:**

Insomnia was slightly more common among mobile phone users in the highest call-time category, but adjustment for the considerably lower RF-EMF exposure from the UMTS than the GSM network suggests that this association is likely due to other factors associated with mobile phone use than RF-EMF. No association was observed for other sleep outcomes. In conclusion, findings from this study do not support the hypothesis that RF-EMF from mobile phone use has long-term effects on sleep quality.

## Highlights

• Above median mobile phone call-time was associated with less sleep disturbance.
• Above 90^th^ percentile mobile phone call time was associated with insomnia.
• Adjustment for network suggests association with insomnia was not due to RF-EMF.
• Other potential explanations may be related to behavior and psychological factors.

## 1 Introduction

Since the introduction of mobile phones, concerns have been raised regarding potential adverse health effects associated with radiofrequency electromagnetic field (RF-EMF) exposure (SCENIHR, 2015). These concerns are accentuated by the rapid increase in the number of mobile phone users worldwide. Mobile phones emit low levels of RF-EMF, and although no biological mechanism for health effects at these low exposure levels has been identified, epidemiological research on such a ubiquitous exposure is necessary to not miss any adverse health effects, as the consequences for public health could be substantial. The prospective Cohort Study of Mobile Phone Use and Health (COSMOS) was initiated to enable investigation of potential effects of RF-EMF exposure on a wide range of health outcomes (Schuz et al., 2011). The main feature of COSMOS is the prospective collection of exposure information and long-term follow-up of health outcomes, to avoid biases inherited in cross-sectional designs and retrospective exposure assessment. This is in line with research agendas issued by the World Health Organization (WHO) and the European Commission Scientific Committee on Emerging and Newly Identified Health Risks (SCENIHR) (SCENIHR, 2009, WHO, 2010).

Sleep disturbance is a commonly reported symptom among persons who perceive themselves as sensitive to low-level electromagnetic field exposure (Roosli et al., 2004). Numerous double-blind provocation studies have consistently shown no short-term effects of RF-EMF exposure on various symptoms, including sleep disturbances (Danker-Hopfe et al., 2010, SCENIHR,2015). However, an effect of RF-EMF on sleep physiology (polysomnography) has been reported in provocation studies, especially on the spectral power of non-REM sleep (SCENIHR,2015, Schmid et al., 2012a), although exposure levels in the study by Schmid et al. were higher (2 W kg^−1^) than levels usually associated with mobile phone use (Schmid et al., 2012a). Findings from studies assessing the effects of RF-EMF on sleep parameters related with the sleep macrostructure are less consistent and encompass both statistically improved and deteriorated sleep (Danker-Hopfe et al., 2011, Danker-Hopfe et al., 2016). The possible clinical significance of the observed physiological changes is unclear and double-blind provocation studies found no effect on self-reported sleep quality or well-being the morning after exposure to RF fields before or during the night (Lowden et al., 2011, Schmid et al., 2012a). However, less data are available on potential effects on sleep quality from long-term RF-EMF exposure.

The few previous prospective epidemiological studies of mobile phone use and sleep quality have been relatively small and results are inconsistent (Cho et al., 2016, Mohler et al., 2012, Thomee et al., 2011, Tokiya et al., 2017). Two of the studies used mobile phone call-time as an indicator of RF-EMF exposure (Cho et al., 2016, Mohler et al., 2012), while the other studies also included other aspects of mobile phone use such as texting when assessing amount of use (Thomee et al., 2011, Tokiya et al., 2017). Recent research on mobile phone use and sleep quality has to an increasing degree focused on behavioral aspects of mobile phone use rather than exposure to RF-EMF: it has been reported that “problematic” mobile phone use (addiction) may affect sleep time, sleep quality, and may cause sleep difficulties (De-Sola Gutierrez et al., 2016, Thomee, 2018).

The lack of support for an acute effect of RF-EMF on wellbeing and sleep does not preclude long-term effects after regular exposure to RF-EMF from mobile phones, especially as the clinical significance of the effects on sleep EEG is unknown. Therefore, the aim of the current study was to investigate long-term effects of RF-EMF exposure from mobile phone use on different sleep outcomes among the Swedish and Finnish participants in the COSMOS study four years after enrollment into the study. Exposure assessment through operator-recorded mobile phone call-time, combined with information on the mobile phone network allows a distinction between mobile phone use behavior and RF-EMF exposure. We have previously reported on long-term follow-up of headaches and hearing impairment (Auvinen et al., 2019).

## 2 Methods

### 2.1 Material

The COSMOS study was initiated to evaluate a broad range of health outcomes in relation to RF-EMF exposure from mobile phone use. The international collaboration includes Denmark, Finland, France, the Netherlands, Sweden and the UK. The current study is based on data from Sweden and Finland, the first two countries to have completed the 4-year follow-up assessment. Study participants were recruited among customers of mobile phone operators in Sweden (in 2008 and 2009) and Finland (in 2009–2011). Stratified sampling by age and amount of mobile phone use was performed to oversample the younger low users and older heavy users, and thereby increase statistical power. More details regarding the recruitment have previously been published (Schuz et al., 2011). In short, over 250,000 and 160,000 individuals were invited to participate in the study in Sweden and Finland, respectively: 50,736 and 13,070 participants filled in the baseline questionnaire and provided informed consent for access to mobile phone operator data. In the Swedish study, the target population was in the age range 18-65 years, but some answered the questionnaire after having turned 66 years. In Finland, the upper age range was 69 years. At baseline, questionnaire information regarding self-reported mobile phone use history, phone numbers of currently used mobile phones, use of hands free devices, potential confounders, and different health outcomes (including sleep outcomes) was collected. In addition, operator recorded mobile phone use was collected for a three-month period at baseline.

The analyses presented here are restricted to participants, aged 18–66 years, who had operator data for all the phone numbers (one or two numbers) listed in the baseline survey (n = 32,286 in Sweden, n = 8186 in Finland), filled in both the baseline and the follow-up questionnaire (n = 22,487 in Sweden, n = 3765 in Finland), did not have missing information about the use of hands-free devices at baseline and did not let other people use their phones often (n = 21,049 in Sweden; n = 3120 in Finland).

### 2.2 Exposure assessment

The exposure of interest in the current study was weekly call-time at baseline estimated from the operator-recorded data (both incoming and outgoing calls). For each call, we also had information regarding network type: Global System for Mobile Communications (GSM) and Universal Mobile Telecommunications System (UMTS) based on the first base station to which the call was connected. Total call-time was estimated by combining call-time in GSM and UMTS networks, while in a separate analysis we divided UMTS call-time by a factor of 150 in order to take into account the difference in output power between these two networks (Auvinen et al., 2019). We also evaluated the effect of call-time on GSM and UMTS networks separately. Call-time in an unknown network was imputed using the individual known ratio of GSM/UMTS call-time. As base station used may shift during a call, we also conducted a sensitivity analysis restricted to participants who only had calls registered on a GSM network, assuming that this subgroup would be less likely to have had any UMTS call-time. This restriction was made to reduce misclassification of network and focus the analysis on the network with the highest RF-EMF exposure.

Self-reported information regarding use of hands-free devices at baseline (“never/almost never”, “less than half of the time”, “approximately half of the time”, “more than half of the time”, “always/almost always”) was used to subtract the proportion of call-time when using a hands-free device from the total call-time. The proportions of subtracted call-time were for each hands-free use category, respectively, 5%, 10%, 25%, 35%, and 50%, based on findings from a previous study (Goedhart et al., 2015). The total call-time was categorized into four groups according to the percentile distribution: <50th percentile (<72 min per week), 50th-74th percentile (72–163 min per week), 75th-89th percentile (164–257 min per week), and ≥90th percentile (≥258 min). In a sensitivity analysis, the hands-free call-time was not deducted, to assess to what degree findings were influenced by the self-reported use of hands-free devices.

Analyses regarding mobile phone use before study entry were performed using self-reported information on starting year of regular mobile phone use (at least once per week) and self-reported amount of mobile phone and hands-free devices use for specific years (2005, 2000, 1995, 1990, 1985).

### 2.3 Sleep outcomes

Sleep outcomes at baseline and follow-up were defined using the 12-item Medical Outcome Study (MOS) sleep scale (Spritzer and Hays, 2003). Each answer is scored from 0 to 100 and all questions in the MOS sleep inventory refer to the 4 weeks preceding the survey. The *a priori* chosen main outcome was a modified version of the MOS sleep disturbance scale. In this modified scale, the question regarding sleep latency was not considered, since we did not want to mix symptoms related to sleep initiation with the ones associated with disturbed sleep. Secondary analyses were performed on other MOS sleep outcomes: daytime somnolence and sleep adequacy. The daytime somnolence scale used here differs from the original MOS scale, since the question regarding naps during the day was not considered; napping may be due not only to sleepiness (replacement napping), but also to pleasure (appetitive napping) (Evans et al., 1977). Scale scores represent the average for all items in the scale that the participants answered, and except for sleep adequacy, a higher score indicates more sleep problems. Sleep latency was analyzed as a dichotomous outcome, using more than 30 min as the cut-off value (Edinger et al., 2004).

An insomnia indicator was defined from the three questions used to compute the modified sleep disturbance scale (“how often did you feel that your sleep was not quiet (moving restlessly, feeling tense, speaking, etc., while sleeping)?”, “how often did you have trouble falling asleep?”, and “how often did you awaken during your sleep time and have trouble falling asleep again?”) combined with an additional fourth question on daytime consequences (“How often did you feel drowsy during the day?”). An answer of “A good part of the time” or more often, to at least one of the first three questions combined with an answer of “A good part of the time” or more often to the question on daytime consequences was used to define the presence of insomnia symptoms, from here on referred to as “insomnia”. The rationale behind this indicator was to mimic the diagnostic criteria for insomnia (Akerstedt et al., 2008, Buysse, 2013, Sateia, 2014). The list of the MOS-12 items used to estimate the different sleep scales is reported in Supplementary Table A.1.

### 2.4 Statistical analysis

The internal consistency of the modified sleep scales used in this study (sleep disturbance and daytime somnolence) was compared to the respective original scale using Cronbach’s alpha. Logistic and linear regression models were used to estimate the effect of operator-recorded mobile phone use (average weekly call-time) at baseline on different sleep outcomes at follow-up. For continuous outcomes (sleep disturbance, daytime somnolence, and sleep adequacy), the sleep score at baseline was included in the model as a potential confounder. When analyzing insomnia and sleep latency (>30 min), the logistic regression model was restricted to individuals who did not have the outcome of interest at baseline. In a sensitivity analysis, we excluded participants who reported at follow-up being woken at night by calls or text messages on their mobile phone. This analysis was performed only on Swedish participants, as this information was not included in the Finnish follow-up questionnaire. Potential confounders decided *a priori* to be included in the regression models were age, gender, country, current smoking, alcohol consumption, body mass index, educational level, weekly headache, mental and physical health score (SF-12), and diagnosis of depression: information about these confounders was collected at baseline. Additional adjustment for cordless phone use at baseline did not change the results (self-reported, categorized as non-regular use (<1 call/week), regular use <1 h/week, and ≥1 h/week). All statistical analyses were performed using Stata 14.2.

## 3 Results

Characteristics of the 24,169 Swedish and Finnish participants included in the study, stratified by amount of mobile phone use, are reported in Table 1. More women than men participated in the study (55% vs 45%) and the large majority of the participants (87%) were recruited in Sweden. Men and young individuals had a slightly higher mobile phone call-time compared to women and older participants (Table 1). At baseline, the prevalence of insomnia was 9%, and 16% reported sleep latency >30 min (not in table). After restriction to individuals who did not report the outcome at baseline, the prevalence of insomnia and sleep latency at follow-up was 6% and 8%, respectively. The internal consistency of the modified sleep disturbance and daytime somnolence scales was similar to that observed for the original scales (Cronbach alpha 0.80 vs 0.83 for sleep disturbance, 0.73 vs 0.68 for daytime somnolence). Analyses of known factors associated with worse sleep outcomes are shown in Supplementary Table A.2, where we confirmed associations with female sex, headaches, depression, alcohol consumption, and smoking. There were some differences between countries in the prevalence of the sleep outcomes at follow-up, especially for sleep disturbance which was more common in Finland, and insomnia which was more common in Sweden (Supplementary Table A.2).

**Table 1:** Characteristics of the study participants and sleep outcomes according to amount of mobile phone use at baseline.

	Average weekly call duration at baseline				
	<72 min	72–163 min	164–257 min	≥258 min	Total
*Gender*^1^					
Men	5013 (47%)	2764 (26%)	1745 (16%)	1238 (12%)	10,760 (45%)
Women	7016 (52%)	3322 (25%)	1884 (14%)	1187 (9%)	13,409 (55%)

*Age*^1^					
18–29	1820 (45%)	1050 (26%)	708 (17%)	499 (12%)	4077 (17%)
30–39	2406 (53%)	1095 (24%)	611 (13%)	471 (10%)	4583 (19%)
40–49	2620 (50%)	1326 (25%)	730 (14%)	547 (10%)	5223 (22%)
50–59	2693 (45%)	1588 (27%)	1040 (17%)	650 (11%)	5971 (25%)
60–66	2490 (58%)	1027 (24%)	540 (13%)	258 (6%)	4315 (18%)

*Country*^1^					
Finland	1286 (41%)	1023 (33%)	483 (16%)	328 (11%)	3120 (13%)
Sweden	10,743 (51%)	5063 (24%)	3146 (15%)	2097 (10%)	21,049 (87%)

*Sleep outcomes at follow-up*					
Sleep disturbance^2^	21.8 (19.8)	21.6 (20.3)	21.5 (20.6)	22.3 (21.8)	21.8 (20.3)
Daytime somnolence^2^	18.2 (17.0)	19.1 (17.7)	19.7 (18.3)	20.7 (19.5)	18.9 (17.7)
Sleep adequacy^2^	60.0 (26.5)	58.3 (27.1)	58.4 (27.0)	56.1 (27.8)	59.0 (26.9)
Insomnia^1^,^3^	585 (5%)	329 (6%)	204 (6%)	158 (7%)	1 276 (6%)
Sleep latency > 30 min^1^,^3^	829 (8%)	394 (8%)	206 (7%)	184 (9%)	1 613 (8%)

Total	12,029 (50%)	6086 (25%)	3629 (15%)	2425 (10%)	24,169

1 Numbers reported indicate number of individuals and prevalence.

2 Numbers reported indicate mean and standard deviation.

3 Restricted to individuals who did not report the outcome at baseline.

Results from linear and logistic regression analyses on long-term effects of mobile phone use are shown in Table 2. In analyses controlled for potential confounders, mobile phone users in the highest call-time category (top decile, >258 min/week) had, on average, a somewhat lower sleep disturbance score at follow-up compared to those 50% with the lowest call-time (regression coefficient, β −0.57, 95% CI −1.30, 0.15). While no association was found between mobile phone call-time and daytime somnolence, mobile phone users in the highest call-time category had less adequate sleep (β −0.83, 95% CI −1.84, 0.17), although not statistically significant, and no trend with call-time was observed. Moreover, inconsistent findings were observed in the two countries (Table 3), although the differences were not statistically significant.

**Table 2:** Effects of mobile phone use at baseline (weekly minutes of conversation) on different indicators of sleep quality at follow-up.

	Adjusted model^1^	Adjusted model^2^
Sleep outcome	β (95% CI)	β (95% CI)
*Sleep disturbance*		
<72 min	0.0 (Ref)	0.0 (Ref)
72–163 min	−0.18 (−0.69, 0.34)	−0.30 (−0.81, 0.22)
164–257 min	−0.78 (−1.40, −0.16)	−1.04 (−1.66, −0.43)
≥258 min	−0.22 (−0.95, 0.51)	−0.57 (−1.30, 0.15)

*Sleep adequacy*		
<72 min	0.0 (Ref)	0.0 (Ref)
72–163 min	−0.85 (−1.57, −0.14)	−0.84 (−1.55, −0.13)
164–257 min	−0.26 (−1.12, 0.61)	0.04 (−0.81, 0.90)
≥258 min	−1.34 (−2.35, −0.32)	−0.83 (−1.84, 0.17)

*Daytime somnolence*		
<72 min	0.0 (Ref)	0.0 (Ref)
72–163 min	0.19 (−0.27, 0.65)	0.16 (−0.30, 0.61)
164–257 min	0.47 (−0.09, 1.02)	0.25 (−0.29, 0.80)
≥258 min	0.62 (−0.03, 1.28)	0.27 (−0.38, 0.91)

	**OR (95% CI)**	**OR (95% CI)**
*Insomnia*^3^		
<72 min	1.0 (Ref)	1.0 (Ref)
72–163 min	1.13 (0.98–1.30)	1.08 (0.93–1.25)
164–257 min	1.18 (0.99–1.39)	1.07 (0.90–1.27)
≥258 min	1.43 (1.19–1.73)	1.24 (1.03–1.51)

*Sleep latency > 30 min*^3^		
<72 min	1.0 (Ref)	1.0 (Ref)
72–163 min	0.93 (0.82–1.06)	0.91 (0.80–1.03)
164–257 min	0.83 (0.71–0.97)	0.77 (0.65–0.91)
≥258 min	1.19 (1.01–1.41)	1.11 (0.93–1.33)

1 Adjusted for age, gender, country, and sleep outcome at baseline.

2 Adjusted for age, gender, country, sleep outcome at baseline, current smoking, alcohol consumption, body mass index, educational level, weekly headache, mental and physical health score (SF-12), and diagnosis of depression.

3 Restricted to individuals who did not report the outcome at baseline (2200 participants reported insomnia, and 4148 reported sleep latency > 30 min at baseline).

**Table 3:** Effects of mobile phone use at baseline (weekly minutes of conversation) on different indicators of sleep quality at follow-up stratified by country and gender.

	Sweden	Finland	Men	Women
Sleep outcome	β (95% CI)^1^	β (95% CI)^1^	β (95% CI)^1^	β (95% CI)^1^
*Sleep disturbance*				
<72 min	0.0 (Ref)	0.0 (Ref)	0.0 (Ref)	0.0 (Ref)
72–163 min	−0.44 (−1.00, 0.12)	0.58 (−0.64, 1.80)	−0.33 (−1.04, 0.37)	−0.19 (−0.92, 0.54)
164–257 min	−1.07 (−1.74, −0.39)	−0.54 (−2.10, 1.02)	−1.61 (−2.44, −0.78)	−0.39 (−1.29, 0.51)
≥258 min	−0.34 (−1.13, 0.45)	−1.78 (−3.61, 0.04)	−0.40 (−1.35, 0.54)	−0.62 (−1.71, 0.47)

*Sleep adequacy*				
<72 min	0.0 (Ref)	0.0 (Ref)	0.0 (Ref)	0.0 (Ref)
72–163 min	−1.10 (−1.88, −0.33)	0.50 (−1.27, 2.26)	−0.47 (−1.51, 0.56)	−1.12 (−2.09, −0.15)
164–257 min	0.01 (−0.91, −0.94)	−0.14 (−2.40, 2.12)	0.99 (−0.23, 2.21)	−0.87 (−2.06, 0.33)
≥258 min	−1.07 (−2.16, 0.02)	0.40 (−2.24, 3.03)	−0.56 (−1.96, 0.83)	−0.99 (−2.44, 0.47)

*Daytime somnolence*				
<72 min	0.0 (Ref)	0.0 (Ref)	0.0 (Ref)	0.0 (Ref)
72–163 min	0.30 (−0.20, 0.79)	−0.64 (−1.78, 0.49)	0.19 (−0.46, 0.84)	0.04 (−0.59, 0.67)
164–257 min	0.35 (−0.24, 0.94)	−0.20 (−1.65, 1.25)	−0.51 (−1.27, 0.25)	0.87 (0.09, 1.65)
≥258 min	0.32 (−0.37, 1.02)	−0.00 (−1.70, 1.69)	0.18 (−0.69, 1.05)	0.27 (−0.67, 1.21)

	**OR (95% CI)**^1^	**OR (95% CI)**^1^	**OR (95% CI)**^1^	**OR (95% CI)**^1^

Insomnia^2^	n = 1137	n = 139	n = 462	n = 814

<72 min	1.0 (Ref)	1.0 (Ref)	1.0 (ref)	1.0 (ref)
72–163 min	1.10 (0.94–1.28)	0.96 (0.63–1.47)	0.94 (0.73–1.20)	1.16 (0.97–1.40)
164–257 min	1.10 (0.91–1.32)	0.93 (0.54–1.60)	0.92 (0.69–1.23)	1.16 (0.93–1.46)
≥258 min	1.35 (1.11–1.66)	0.60 (0.30–1.21)	1.25 (0.93–1.68)	1.21 (0.94–1.57)

Sleep latency > 30 min^2^	n = 1406	n = 207	n = 559	n = 1054
<72 min	1.0 (Ref)	1.0 (Ref)	1.0 (ref)	1.0 (ref)
72–163 min	0.88 (0.76–1.01)	1.04 (0.73–1.49)	0.78 (0.63–0.97)	1.00 (0.85–1.18)
164–257 min	0.74 (0.62–0.89)	0.89 (0.56–1.42)	0.60 (0.45–0.80)	0.90 (0.73–1.11)
≥258 min	1.03 (0.85–1.25)	1.70 (1.07–2.70)	1.04 (0.80–1.36)	1.18 (0.93–1.48)

1 Adjusted for age, gender (in country specific analyses), country (in gender specific analyses), sleep outcome at baseline, current smoking, alcohol consumption, body mass index, educational level, weekly headache, mental and physical health score (SF-12), and diagnosis of depression.

2 Restricted to individuals who did not report the outcome at baseline. In Sweden 1976 participants reported insomnia, and 3412 reported sleep latency >30 min at baseline. Corresponding numbers for Finland was 224 and 556, respectively.

The odds ratio (OR) for > 30 min sleep latency at follow-up was 1.19 (95% CI 1.01–1.41) in the highest call-time decile at baseline, but was close to unity after adjustment for potential confounders (OR 1.11, 95% CI 0.93–1.33) (Table 2). Differences between countries were not statistically significant. The corresponding result for insomnia at follow-up was 1.43 (95% CI 1.19–1.73), which was weakened after adjustment for potential confounders (OR 1.24, 95% 1.03–1.51). A similar finding was observed in a sensitivity analysis, in which use of hands-free devices was not considered (Supplementary Table A.3). The association for insomnia was observed in both men and women but was driven by the Swedish data (Table 3). The difference between countries was statistically significant (p = 0.01). When UMTS call-time was divided by a factor of 150 to take into consideration the lower RF-EMF exposure compared to the GSM network, the OR for insomnia for participants in the highest decile of call-time was 1.09 (95% CI 0.89–1.33) with no trend across exposure categories (Table 4). In analyses stratified by network type (GSM/UMTS), the ORs for insomnia at follow-up for the highest decile of GSM (≥190 min) and UMTS (≥122 min) call-time were of similar magnitude (Table 4). Participants in the highest decile of GSM call-time experienced less sleep disturbance than those in the reference group (β −1.22, 95% CI −1.95, −0.48), while neither GSM or UMTS call-time was associated with the other sleep outcomes (Table 4). When analyses were restricted to participants with calls only on a GSM network (n = 11,676), no adverse effects of call-time on any of the sleep outcomes were observed (Table 4).

**Table 4:** Effects of mobile phone use at baseline (weekly minutes of conversation) in GSM and UMTS systems (first base station) on different sleep outcomes at follow-up.

	Sleep disturbance	Sleep adequacy	Daytime somnolence	Insomnia^1^	Sleep latency > 30 min^1^
Exposure indicator^2^	β (95% CI)^3^	β (95% CI)^3^	β (95% CI)^3^	OR (95% CI)^3^	OR (95% CI)^3^
*All calls, UMTS call time divided by 150*					
<24 min	0.0 (Ref)	0.0 (Ref)	0.0 (Ref)	1.0 (Ref)	1.0 (Ref)
24–84 min	−0.63 (−1.15, −0.10)	−0.19 (−0.91, 0.54)	0.12 (−0.34, 0.58)	1.17 (1.01–1.36)	0.91 (0.79–1.04)
85–189 min	−0.58 (−1.20, 0.04)	−0.33 (−1.19, 0.54)	0.33 (−0.22, 0.88)	1.18 (0.99–1.40)	0.91 (0.78–1.07)
190 + min	−1.22 (−1.95, −0.48)	−0.18 (−1.20, 0.83)	−0.06 (−0.71, 0.59)	1.09 (0.89–1.33)	0.88 (0.73–1.07)

*Time on GSM network*^4^					
<24 min	0.0 (Ref)	0.0 (Ref)	0.0 (Ref)	1.0 (Ref)	1.0 (Ref)
24–84 min	−0.64 (−1.17, −0.11)	−0.16 (−0.90, 0.57)	0.12 (−0.35, 0.59)	1.17 (1.01–1.36)	0.91 (0.80–1.04)
85–189 min	−0.55 (−1.18, 0.07)	−0.44 (−1.30, 0.42)	0.34 (−0.21, 0.89)	1.20 (1.00–1.42)	0.93 (0.79–1.08)
190 + min	−1.24 (−1.99, −0.50)	−0.35 (−1.39, 0.68)	−0.03 (−0.69, 0.64)	1.14 (0.93–1.40)	0.89 (0.74–1.08)

*Time on UMTS network*^4^					
0 min	0.0 (Ref)	0.0 (Ref)	0.0 (Ref)	1.0 (Ref)	1.0 (Ref)
1–34 min	−0.07 (−0.60, 0.46)	−0.37 (−1.10, 0.36)	−0.21 (−0.67, 0.26)	1.06 (0.91–1.23)	1.12 (0.98–1.29)
35–121 min	0.02 (−0.62, 0.66)	−0.85 (−1.74, 0.03)	0.19 (−0.37, 0.76)	1.19 (1.00–1.42)	0.96 (0.81–1.13)
122 + min	0.21 (−0.54, 0.95)	−0.87 (−1.90, 0.17)	0.45 (−0.22, 1.11)	1.21 (0.98–1.48)	1.19 (0.99–1.44)

*Restricted to participants with only GSM call time*^5^					
<24 min	0.0 (Ref)	0.0 (Ref)	0.0 (Ref)	1.0 (Ref)	1.0 (Ref)
24–84 min	−0.71 (−1.54, 0.11)	0.12 (−1.03, 1.27)	0.06 (−0.67, 0.80)	1.20 (0.94–1.54)	0.95 (0.76–1.18)
85–189 min	−0.54 (−1.42, 0.35)	−0.24 (−1.47, 1.00)	0.25 (−0.54, 1.04)	1.12 (0.86–1.46)	0.99 (0.78–1.25)
190 + min	−1.94 (−2.88, −1.00)	0.00 (−1.30, 1.31)	−0.11 (−0.95, 0.73)	1.08 (0.82–1.42)	0.84 (0.65–1.08)

1 Restricted to individuals who did not report the outcome at baseline

2 Categorized according to the 50th, 75th, and 90th percentiles.

3 All analyses were adjusted for age, gender, country, sleep outcome at baseline, current smoking, alcohol consumption, body mass index, educational level, weekly headache, mental and physical health score (SF-12), and diagnosis of depression.

4 GSM and UMTS calls were included in the same regression model.

5 n = 11,676.

Analyses regarding the effect of self-reported lifetime mobile phone use (average lifetime call-time and years of use) showed no clear pattern in relation to sleep outcomes (Supplementary Table A.4). For example, individuals with moderate average lifetime call-time (164–257 min per week) had an increased odds of insomnia at follow-up (OR = 1.47, 95% CI 1.22–1.78), while for the highest decile (≥258 min per week), the risk estimate was lower (OR = 1.17, 95% 0.88–1.57). High average self-reported lifetime mobile phone call-time was associated with sleep latency at follow-up (OR 1.38, 95% CI 1.09–1.74). Number of years of regular mobile phone use was not associated with any of the sleep outcomes (Supplementary Table A.4).

In a sensitivity analysis based only on Swedish participants, individuals reporting insomnia at follow-up were more likely to be woken at night by calls or text messages on their mobile phones at follow-up, compared to those without insomnia (30% vs. 20%). Moreover, 30% of participants in the top decile of call-time at baseline reported that they were woken at night by calls or text messages: among participants in the lowest exposure category (<72 min/week) this proportion was 15%. While in the country-specific analysis, Swedish mobile phone users in the highest category of call-time had an OR of 1.35 (95% CI 1.11–1.66) for insomnia, after restricting the analysis to Swedish participants that were not woken by calls or text messages, a slightly weaker association was observed (OR = 1.20, 95% CI 0.92–1.55) and there was also no clear trend across the exposure categories (Supplementary Table A.5). No effect on the other sleep outcomes was observed (Supplementary Table A.5).

## 4 Discussion

In this prospective cohort study with almost 25,000 participants, operator-recorded mobile phone call-time at baseline was not associated with sleep disturbance at follow-up, the *a priori* defined main outcome. Although effect estimates indicated that mobile phone call-time was associated with less sleep disturbance, there was no consistent exposure response pattern, and wide confidence intervals in the highest exposure category. There was little or no effect also on most of the other studied sleep outcomes. We found an increased prevalence of insomnia at follow-up among participants in the top decile of mobile phone call-time (>258 min/week), but this association diminished after adjustment for the level of RF exposure generated by the UMTS compared to the GSM network. This indicates possible confounding from other factors associated with mobile phone use, as discussed in more detail below.

So far, only few previous prospective cohort studies have investigated the effect of mobile phone use on sleep quality (Cho et al., 2016, Mohler et al., 2012, Thomee et al., 2011, Tokiya et al., 2017). A Swedish study of 4156 young adults (aged 20–24 years) reported cross-sectional associations between high mobile phone use (a combination of calls and SMS message frequency) and sleep disturbances: however, a prospective association was found only among men (Thomee et al., 2011). A Swiss study of 955 participants (aged 30–60 years) with a one-year follow-up found that mobile phone call-time or exposure to environmental RF-EMF were not associated with sleep disturbances or daytime sleepiness (Mohler et al., 2012). A Korean study of 532 individuals followed up for two years after assessment of mobile phone call-time by interviews also failed to find any effect on sleep (Cho et al., 2016). A Japanese study investigating predictors of insomnia among over 3000 high school students reported an increase in onset of insomnia for heavy mobile phone users (>2 h/day) in the senior, but not junior high school group after two years follow-up (Tokiya et al., 2017). Mobile phone use in the Japanese study may have included other activities such as texting or gaming. The Japanese results are partly in agreement with our findings for insomnia, when we considered call-time without adjustment for RF-EMF levels associated with type of network.

Insomnia patients have been shown to have less REM sleep and more frequent micro- and macro-arousals during REM sleep than matched controls. This has led to the REM sleep instability hypothesis of insomnia (Riemann et al., 2012), supported by a meta-analysis of polysomnography studies (Baglioni et al., 2014). Provocation studies have investigated whether RF-EMF exposure is associated with shorter REM sleep duration, but their results are not consistent. While two studies reported an association of RF-EMF exposure with a reduced REM sleep phase (Mann and Roschke, 1996, Schmid et al., 2012b), a recent study found an increase (Danker-Hopfe et al., 2016). Not only are findings from these studies inconsistent but, since they have estimated only the short-term effect of RF-EMF exposure on REM sleep phase, they do not allow conclusions on long-term effects.

In the current study, the association between mobile phone use in the highest category of call-time and insomnia was almost identical, regardless of whether use of hands-free devices was considered or not. Moreover, two hours or more of call-time per week on a UMTS network was associated with a slightly higher increased odds of insomnia compared to more than three hours per week on a GSM network, even though the output power is considerably higher (approximately 150 times) on a GSM than UMTS network. In addition, when we restricted analyses to participants who only had calls on the GSM network, to reduce misclassification of network which may have been caused by a change of base station during a call (since we only had information on network type from the first base station of the call), amount of call-time had no adverse effect on any of the sleep outcomes. These results indicate that the association between mobile phone call-time at baseline and insomnia at follow-up is likely determined by other aspects of mobile phone use than RF-EMF exposure, such as behavioral factors, e.g. addictive mobile phone use, or unmeasured confounding factors. The newer generation’s mobile phone technology is a prerequisite for use of the phone for social media, internet, gaming, and data downloads, i.e. usage where the mobile phone is kept in the hand rather than to the ear.

Available research on mobile phone use and sleep quality suggests that mobile phone use might disrupt sleep through mechanisms that are not related to RF-EMF exposure. In fact, media use before bedtime or after lights out, including mobile phone use, has been associated with sleep loss, poorer sleep quality, and increased tiredness during the day, particularly among adolescents and young adults (Bartel et al., 2015, Kubiszewski et al., 2014, Owens et al., 2014). Moreover, another study showed that mobile phone use before bedtime had a negative impact on sleep outcomes also among adults (Exelmans and Van den Bulck, 2016). A possible explanation for these findings is that blue light emitted by mobile phone screens or tablets, particularly around bedtime, suppresses the secretion of melatonin, a hormone regulating sleep and wakefulness, and hence delaying sleep onset and disrupting sleep (Cajochen et al., 2011, Chellappa et al., 2013, Wood et al., 2013). Mobile phones where blue light can be turned off were introduced to the market only towards the end of the follow-up data collection. However, it is also possible that the activity *per se* may cause arousal decreasing the ability to fall asleep. When we restricted analyses to individuals who were not awaken at night by calls or text messages, the OR for insomnia in the highest call-time category decreased substantially. This suggests that the association between call-time at baseline and insomnia at follow-up in the main analysis could be explained by other factors not related with RF-EMF from mobile phones, such as mobile phone use before or after bedtime. In addition, we did not have access to information about stress and high demands in general, which are potential confounders that may lead to both a high frequency of mobile phone use and insomnia.

## 5 Strength and limitations

Major strengths are the prospective design of this large cohort study, access to participants’ operator data for a three-month period close to the baseline, information on mobile phone network used for each call, and a four-year period between baseline and follow-up. This gave us the opportunity to investigate long-term effects of mobile phone use on sleep quality. Thus, our study was not affected by recall bias, non-differential exposure misclassification was minimized, and type of network allowed us to improve the assessment of RF-EMF exposure compared to previous research where only the call-time has been assessed. In addition, we used a validated instrument to assess sleep outcomes, and could take into consideration sleep outcomes at baseline when assessing effects at follow-up.

We used the 12-item MOS sleep scale both at baseline and at follow-up to evaluate sleep disturbance, insomnia, daytime somnolence, sleep adequacy, and sleep latency. For sleep disturbance and daytime somnolence, we used a modified version of the MOS scales, but sensitivity analysis in which the original MOS scales were used showed almost identical results (data not shown). Moreover, the internal consistency of the modified sleep scales, based on the Cronbach alpha value, was similar to the one obtained for the original scales.

We used an insomnia indicator consistent with the diagnostic criteria for insomnia, although it did not take duration and frequency of insomnia symptoms into consideration (Akerstedt et al., 2008, Buysse, 2013, Sateia, 2014). The dimensions of sleep used to establish the diagnosis of insomnia include difficulties falling asleep, frequent awakenings, early final awakenings, or non-restorative sleep (Buysse, 2013, Sateia, 2014). Sleep questionnaires/indices focusing on insomnia usually include two main dimensions, sleep quality and non-restorative sleep (Akerstedt et al., 2008, Nordin et al., 2013). The first contains items like difficulties falling asleep, frequent awakenings, early morning awakening, and restless sleep, while the second pertains to difficulties awakening, feeling well rested, and having had enough sleep. In the current study, the prevalence of insomnia at baseline was approximately 9%, which is in agreement with previous studies (Buysse, 2013, Mallon et al., 2014). Results from the analyses of factors known to be associated with worse sleep outcomes indicate that the self-reported sleep outcomes used in the study appropriately identified individuals with sleep problems (Franzen and Buysse, 2008, Kelman and Rains, 2005, Middelkoop et al., 1996, Wetter and Young, 1994).

A limitation of the study is that we did not have information about the sleep outcomes or mobile phone use during the follow-up period. Therefore, we could only evaluate the effect of mobile phone use on the prevalence of insomnia at follow-up approximately four years after baseline, rather than on the occurrence of insomnia during the entire follow-up period. Assessing incidence of insomnia in the intervening period would have required repeated contacts with the participants over a four-year period, which was not feasible. Moreover, in the baseline questionnaire, we did not have information on levels of stress and addictive mobile phone use, which might have had an impact on the amount of mobile phone use and sleep quality. Also, there is a slight difference in the calendar period of data collection; a two-year period starting 2008 in Sweden and a three-year period starting 2009 in Finland. Another limitation was the lack of information on network used beyond the first base station of the call, which may have led to misclassification of calls between GSM and UMTS; however, our analyses restricted to participants with only GSM calls listed did not point to any increased risks for those exposures.

## 6 Conclusions

In this prospective cohort study, we found no association between mobile phone call-time at baseline and sleep disturbance at the 4-year follow-up. We found a moderate association in the highest decile of mobile phone call-time at baseline (>258 min/week) with insomnia at follow-up. However, analyses considering the lower RF-EMF exposure from the UMTS compared to the GSM network suggest that this was not due to RF-EMF but likely due to other aspects of mobile phone use. Such factors could be stress and high demands, problematic mobile phone use, displacement of sleep, other behavioral factors, exposure to blue light at bedtime, or other unmeasured confounding factors. Further research is needed to clarify which aspects of mobile phone use are associated with insomnia. In conclusion, findings from this study do not support the hypothesis that RF-EMF exposure from mobile phone use has long-term effects on sleep quality.

## CRediT authorship contribution statement

**Giorgio Tettamanti:** Methodology, Formal analysis, Data curation, Writing - original draft, Writing - review & editing, Visualization. **Anssi Auvinen:** Conceptualization, Methodology, Investigation, Writing - review & editing, Supervision, Funding acquisition. **Torbjörn Åkerstedt:** Methodology, Writing - review & editing. **Katja Kojo:** Investigation, Data curation, Writing - review & editing. **Anders Ahlbom:** Conceptualization, Methodology, Writing - review & editing, Funding acquisition. **Sirpa Heinävaara:** Investigation, Writing - review & editing. **Paul Elliott:** Conceptualization, Methodology, Writing - review & editing. **Joachim Schüz:** Methodology, Writing - review & editing. **Isabelle Deltour:** Methodology, Writing - review & editing. **Hans Kromhout:** Methodology, Writing - review & editing. **Mireille B. Toledano:** Methodology, Writing - review & editing. **Aslak Harbo Poulsen:** Methodology, Writing - review & editing. **Christoffer Johansen:** Methodology, Writing - review & editing. **Roel Vermeulen:** Methodology, Writing - review & editing. **Maria Feychting:** Conceptualization, Methodology, Writing - review & editing, Visualization, Project administration, Funding acquisition. **Lena Hillert:** Conceptualization, Methodology, Investigation, Writing - review & editing, Supervision. **:** .

## Declaration of Competing Interest

The authors declare the following financial interests/personal relationships which may be considered as potential competing interests: M.F. is vice chairman of the International Commission on Non-Ionizing Radiation Protection, an independent body setting guidelines for non-ionizing radiation protection. She has served as advisor to a number of national and international public advisory and research steering groups concerning the potential health effects of exposure to non-ionizing radiation, for example the World Health Organization. H.K. is the chair of the Committee on Electromagnetic Fields of the Health Council of The Netherlands. All other authors have declared no conflict of interest.
