# Supplementary material for: Long-term effect of mobile phone use on sleep quality: Results from the cohort study of mobile phone use and health (COSMOS)
Source: Environ Int. 2020 Jul;140:105687. doi: 10.1016/j.envint.2020.105687 (PMC7272128; doi:10.1016/j.envint.2020.105687)
Supplement: Supplementary data 1 [file mmc1.docx]

Supplementary Table A.1: The Medical Outcome Study 12-item sleep inventory and sleep indicators used in the study

|  | Modified sleep disturbance scale | MOS Sleep adequacy (SLPA2) | Modified daytime somnolence scale | Insomnia^1^ | Sleep latency |
| --- | --- | --- | --- | --- | --- |
| 1) How long did it usually take for you to fall asleep during the past 4 weeks? |  |  |  |  | X |
| 2) On the average, how many hours did you sleep each night during the past 4 weeks? |  |  |  |  |  |
| **How often during the past 4 weeks did you…** | | | | |  |
| 3) feel that your sleep was not quiet (moving restlessly, feeling tense, speaking, etc., while sleeping)? | X |  |  | X |  |
| 4) get enough sleep to feel rested upon waking in the morning? |  | X |  |  |  |
| 5) awaken short of breath or with a headache? |  |  |  |  |  |
| 6) feel drowsy or sleepy during the day? |  |  | X | X |  |
| 7) have trouble falling asleep? | X |  |  | X |  |
| 8) awaken during your sleep time and have trouble falling asleep again? | X |  |  | X |  |
| 9) have trouble staying awake during the day? |  |  | X |  |  |
| 10) snore during your sleep? |  |  |  |  |  |
| 11) take naps (5 minutes or longer) during the day? |  |  |  |  |  |
| 12) get the amount of sleep you needed? |  | X |  |  |  |

1 Insomnia was defined as an answer of “A good bit of the time”, or more often, to at least one of questions 3, 7, or 8 combined with an answer of “A good bit of the time” or more often to question 6

Supplementary Table A.2: Effects of gender, alcohol intake, body mass index, current smoking, weekly headache and depression at baseline on different sleep outcomes at follow-up

|  | **Sleep disturbance** | **Sleep adequacy** | **Daytime somnolence** | **Insomnia^1^** | **Sleep latency > 30 min^1^** |
| --- | --- | --- | --- | --- | --- |
| **Exposure indicator** | **β (95% CI)^2^** | **β (95% CI)^2^** | **β (95% CI)^2^** | **OR (95% CI)^2^** | **OR (95% CI)^2^** |
| **Gender** |  |  |  |  |  |
| Men | 0.0 (Ref) | 0.0 (Ref) | 0.0 (Ref) | 1.0 (Ref) | 1.0 (Ref) |
| Women | 2.12 (1.67, 2.58) | -1.82 (-2.45, -1.19) | -0.59 (-0.99, -0.19) | 1.12 (0.98-1.28) | 1.45 (1.28-1.63) |
|  |  |  |  |  |  |
| **Alcohol intake** |  |  |  |  |  |
| 0-1 drinks/week | 0.0 (Ref) | 0.0 (Ref) | 0.0 (Ref) | 1.0 (Ref) | 1.0 (Ref) |
| 2-4 drinks/week | 0.25 (-0.31, 0.82) | 0.48 (-0.31, 1.27) | -0.43 (-0.94, 0.07) | 0.91 (0.78-1.07) | 1.06 (0.92-1.22) |
| 5-8 drinks/week | 0.76 (0.11, 1.42) | -0.21 (-1.12, 0.70) | -0.34 (-0.92, 0.24) | 0.98 (0.81-1.18) | 1.01 (0.85-1.19) |
| 9+ drinks/week | 1.63 (0.97, 2.28) | -0.20 (-1.10, 0.70) | -0.16 (-0.73, 0.42) | 1.20 (1.00-1.43) | 1.21 (1.03-1.43) |
|  |  |  |  |  |  |
| **Body mass index** |  |  |  |  |  |
| Underweight | 0.58 (-1.28, 2.43) | 0.35 (-2.23, 2.92) | 1.69 (0.05, 3.34) | 1.01 (0.63-1.61) | 1.37 (0.91-2.07) |
| Normal weight | 0.0 (Ref) | 0.0 (Ref) | 0.0 (Ref) | 1.0 (Ref) | 1.0 (Ref) |
| Overweight | 0.06 (-0.41, 0.53) | -0.27 (-0.92, 0.39) | 0.05 (-0.37, 0.47) | 1.01 (0.88-1.16) | 0.98 (0.87-1.11) |
| Obese | 0.52 (-0.14, 1.19) | -0.30 (-1.23, 0.62) | 0.87 (0.28, 1.46) | 1.14 (0.95-1.36) | 1.14 (0.97-1.34) |
|  |  |  |  |  |  |
| **Current smoking** |  |  |  |  |  |
| No | 0.0 (Ref) | 0.0 (Ref) | 0.0 (Ref) | 1.0 (Ref) | 1.0 (Ref) |
| Yes | 0.63 (-0.04, 1.31) | -1.25 (-2.18, -0.31) | 1.25 (0.65, 1.84) | 1.41 (1.19-1.67) | 1.35 (1.16-1.59) |
|  |  |  |  |  |  |
| **Weekly headache** |  |  |  |  |  |
| No | 0.0 (Ref) | 0.0 (Ref) | 0.0 (Ref) | 1.0 (Ref) | 1.0 (Ref) |
| Yes | 1.98 (1.38, 2.57) | -3.15 (-3.97, -2.33) | 2.05 (1.52, 2.57) | 1.54 (1.34-1.77) | 1.24 (1.08-1.42) |
|  |  |  |  |  |  |
| **Depression** |  |  |  |  |  |
| No | 0.0 (Ref) | 0.0 (Ref) | 0.0 (Ref) | 1.0 (Ref) | 1.0 (Ref) |
| Yes | 2.54 (1.88, 3.20) | -2.51 (-3.43, -1.59) | 2.35 (1.76, 2.94) | 1.37 (1.17-1.61) | 1.55 (1.34-1.80) |
|  |  |  |  |  |  |
| **Country** |  |  |  |  |  |
| Sweden | 0.0 (Ref) | 0.0 (Ref) | 0.0 (Ref) | 1.0 (Ref) | 1.0 (Ref) |
| Finland | 1.19 (0.55, 1.83) | -0.63 (-1.52, 0.25) | 1.37 (0.80, 1.94) | 0.76 (0.63-0.93) | 0.95 (0.81-1.12) |

1 Restricted to individuals who did not report the outcome at baseline

2 Adjusted for age, gender, country, sleep outcome at baseline, current smoking, alcohol consumption, body mass index, educational level, weekly headache, mental and physical health score (SF-12), and diagnosis of depression

Supplementary Table A.3: Effects of mobile phone use baseline (weekly minutes of conversation) on different indicators of sleep quality at follow-up without adjustment for reported proportion of hands free call time

| **Sleep outcome** | **β (95% CI)^1^** |
| --- | --- |
| **Sleep disturbance** |  |
| <72 min | 0.0 (Ref) |
| 72-163 min | -0.33 (-0.86,0.21) |
| 164-257 min | -1.00 (-1.61,-0.39) |
| ≥258 min | -0.75 (-1.36,-0.13) |
| **Sleep adequacy** |  |
| <72 min | 0.0 (Ref) |
| 72-163 min | -0.78 (-1.53,-0.04) |
| 164-257 min | -0.52 (-1.37,0.32) |
| ≥258 min | -0.42 (-1.28,0.43) |
| **Daytime somnolence** |  |
| <72 min | 0.0 (Ref) |
| 72-163 min | -0.05 (-0.52,0.43) |
| 164-257 min | 0.47 (-0.07,1.01) |
| ≥258 min | 0.14 (-0.41,0.68) |
|  | **OR (95% CI)** |
| **Insomnia^2^** |  |
| <72 min | 1.0 (Ref) |
| 72-163 min | 1.04 (0.89,1.21) |
| 164-257 min | 1.06 (0.89,1.26) |
| ≥258 min | 1.22 (1.04,1.44) |
| **Sleep latency > 30 min^2^** |  |
| <72 min | 1.0 (Ref) |
| 72-163 min | 0.94 (0.82-1.08) |
| 164-257 min | 0.75 (0.63-0.88) |
| ≥258 min | 0.99 (0.85-1.16) |

1 Adjusted for age, gender, country, sleep outcome at baseline, current smoking, alcohol consumption, body mass index, educational level, weekly headache, mental and physical health score (SF-12), and diagnosis of depression

2 Restricted to individuals who did not report the outcome at baseline

Supplementary Table A.4: Effect of self-reported lifetime mobile phone use at baseline (weekly minutes of conversation and years of use) on different indicators of sleep quality at follow-up

|  | **Sleep disturbance** | **Sleep adequacy** | **Daytime somnolence** | **Insomnia^1^** | **Sleep latency > 30 min^1^** |
| --- | --- | --- | --- | --- | --- |
| **Exposure indicator** | **β (95% CI)^2^** | **β (95% CI)^2^** | **β (95% CI)^2^** | **OR (95% CI)^2^** | **OR (95% CI)^2^** |
| **Average historical mobile phone use** |  |  |  |  |  |
| <72 min | 0.0 (Ref) | 0.0 (Ref) | 0.0 (Ref) | 1.0 (Ref) | 1.0 (Ref) |
| 72-163 min | -0.02 (-0.53,0.50) | -0.39 (-1.11,0.32) | 0.49 (0.03,0.95) | 1.24 (1.07,1.43) | 0.96 (0.84-1.10) |
| 164-257 min | 0.17 (-0.55,0.90) | -0.57 (-1.58,0.43) | 1.12 (0.48,1.77) | 1.47 (1.22,1.78) | 0.93 (0.77-1.13) |
| 258+ min | -0.51 (-1.52,0.49) | -0.21 (-1.61,1.19) | 0.81 (-0.09,1.70) | 1.17 (0.88,1.57) | 1.38 (1.09-1.74) |
| **Years of use** |  |  |  |  |  |
| 0-5 years of use | 0.75 (-0.02,1.52) | 1.69 (0.61,2.76) | 0.64 (-0.05,1.32) | 1.21 (0.98,1.49) | 1.07 (0.88-1.29) |
| 6-10 years of use | 0.0 (Ref) | 0.0 (Ref) | 0.0 (Ref) | 1.0 (Ref) | 1.0 (Ref) |
| 11-15 years of use | 0.57 (0.04,1.11) | -0.45 (-1.19,0.29) | -0.01 (-0.49,0.46) | 1.06 (0.91,1.23) | 1.06 (0.93-1.21) |
| 16+ years of use | 0.09 (-0.58,0.76) | 0.07 (-0.86,0.99) | 0.07 (-0.52,0.66) | 1.09 (0.89,1.33) | 0.87 (0.73-1.03) |

1 Restricted to individuals who did not report the outcome at baseline

2 Adjusted for age, gender, country, sleep outcome at baseline, current smoking, alcohol consumption, body mass index, educational level, weekly headache, mental and physical health score (SF-12), and diagnosis of depression

Supplementary Table A.5: Sensitivity analyses restricted to participants that were not awakened at night by a call or text message at follow-up

|  | **Sleep disturbance** | **Sleep adequacy** | **Daytime somnolence** | **Insomnia^1^** | **Sleep latency > 30 min^1^** |
| --- | --- | --- | --- | --- | --- |
|  | **β (95% CI)^2^** | **β (95% CI)^2^** | **β (95% CI)^2^** | **OR (95% CI)^2^** | **OR (95% CI)^2^** |
| **Not awaken by calls or text messages^1^** |  |  |  |  |  |
| <72 min | 0.0 (Ref) | 0.0 (Ref) | 0.0 (Ref) | 1.0 (Ref) | 1.0 (Ref) |
| 72-163 min | -0.35 (-0.97, 0.27) | -1.26 (-2.13, -0.38) | 0.24 (-0.30, 0.79) | 1.16 (0.96-1.39) | 0.92 (0.78-1.08) |
| 164-257 min | -1.21 (-1.96, -0.46) | 0.29 (-0.78, 1.35) | 0.30 (-0.36, 0.96) | 1.15 (0.92-1.43) | 0.65 (0.52-0.81) |
| 258+ min | -0.77 (-1.69, 0.15) | -0.37 (-1.66, 0.93) | -0.23 (-1.03, 0.58) | 1.20 (0.92-1.55) | 0.95 (0.75-1.21) |

1 Analyses restricted only to Swedish participants
